# Enhancing children’s nutrition: the influence of rural household technology under China’s home appliances going to the countryside policy

**DOI:** 10.3389/fnut.2024.1335200

**Published:** 2024-03-21

**Authors:** Mingling Du, Junhui Shi, Songping Shi, Fang Wang

**Affiliations:** College of Management, Sichuan Agricultural University, Chengdu, China

**Keywords:** HAGC policy, nutrition intake, household technology, child malnutrition, home appliances

## Abstract

This study explores the influence of household technological advancements on children’s nutritional intake, specifically within the context of the Chinese government’s “Home Appliances Going to the Countryside” (HAGC) initiative. Utilizing data from the China Health and Nutrition Surveys of 2006, 2009, and 2011, we employed a Propensity Score Matching Difference-in-Differences (PSM-DID) framework to ascertain the repercussions of enhanced household technology on the dynamics of children’s nutritional consumption patterns. Our analysis reveals that the HAGC-inspired integration of household appliances, including color televisions, washing machines, and refrigerators, has beneficially reshaped the nutritional consumption patterns of children, with a pronounced effect among female children. This finding remains consistent even when employing alternate methodological robustness tests. A deeper examination of the HAGC policy’s mechanisms underscores the pivotal roles of parental time allocation, improved food storage capabilities, and augmented information accessibility as significant drivers bolstering children’s nutritional intake. These insights bear considerable significance for strategizing interventions aimed at elevating the nutritional wellbeing of children in rural settings, offering valuable input for shaping public health policies tailored for such demographies.

## 1 Introduction

Child malnutrition remains pervasive in rural sectors of developing nations, warranting urgent global intervention. Current estimates suggest a staggering 10 million child fatalities between 2019 and 2030, with malnutrition contributing to 45% of these annual child deaths (1). Malnutrition, encompassing both undernutrition and overnutrition, stems from inadequate energy and nutrient consumption (2). Such deficiencies pose profound threats to the health and developmental trajectories of children in these underserved regions. Alarmingly, numerous families in these areas remain unaware or indifferent to this escalating issue, despite its profound implications on cognitive development, academic achievements, and future productivity (3, 4). The ripple effects of malnutrition also compromise children’s latent potential. In its extreme manifestations, malnutrition exacerbates susceptibility to infections, escalates morbidity rates, and even precipitates death (5, 6). Addressing rural child nutrition transcends individual welfare, resonating with the broader stability and sustainable evolution of global rural communities. As the world’s preeminent developing nation, China too grapples with significant nutritional challenges among its rural youth. With a majority of malnourished children hailing from rural hinterlands (7), the pivotal question centers on leveraging governmental initiatives to enhance the nutritional wellbeing of China’s rural children.

The 20th-century witnessed a seismic shift in household technology, fuelling an insatiable global appetite for major appliances, especially within burgeoning economies. This technological metamorphosis has seamlessly woven convenience, intelligence, and connectivity into our domestic fabric. The omnipresence of technology in contemporary households is manifested across diverse domains, including interactive entertainment, operational efficiency, and resource conservation (8). Seminal innovations like microwaves and refrigerators, synergized with the surging availability of ready-to-cook foods, have dramatically streamlined culinary chores (9). Television, a cornerstone of modern household technology, emerges as a pivotal conduit for both entertainment and knowledge dissemination (10). Recognizing the traditionally entrenched roles of women in domestic orchestration, technological advancements alleviate their domestic burdens, bestowing them greater temporal autonomy (11). Yet, the nexus between technological domestic enhancements and health metrics remains relatively uncharted. This study endeavors to bridge this knowledge chasm, scrutinizing the ramifications of household technological progress on child health metrics, emphasizing nutritional outcomes in developing regions. Our insights hope to illuminate the instrumental role of technology in uplifting living standards and health paradigms in marginalized rural precincts.

This study aims to discern the impact of advancements in household technology, arising from the deployment of the “Home Appliances Going to the Countryside” (HAGC) policy, on the nutritional intake of children in rural areas. Introduced amidst the global financial crisis, the HAGC policy was operational until 2013, spanning 4 years. Designed to incentivize farmers to adopt household appliances, it subtly transformed the dietary habits in these regions. As a result of HAGC’s influence, rural families increasingly integrated household appliances, particularly color TVs, washing machines, and refrigerators, into their daily lives. The adoption of these appliances afforded busy farmers the luxury of time, enabling them to invest greater effort in preparing nutritionally balanced meals for their children, leading to a richer dietary composition. In our analysis, we leverage data from the China Health and Nutrition Survey (CHNS) to establish a causal link between household technology enhancements and children’s nutritional intake, utilizing the propensity score matched difference method (PSM-DID). To reinforce the validity of our primary model’s findings, we also performed robustness tests, incorporating alternative matching techniques and explanatory variables. Recognizing disparities in nutritional intake patterns between genders, we also undertook heterogeneity analyses. Furthermore, we delved into the underlying mechanisms through which household technology advancements bolster children’s nutritional intake.

The marginal contributions of this study are as follows: (1) An extensive probe into child malnutrition worldwide, especially within developing nations, emphasizing its profound repercussions on child health and development and advocating for amplified nutritional support for rural progeny. (2) An insightful inquiry into China’s approach—being a predominant developing nation—to mitigate malnutrition among its rural children, elucidating the symbiotic relationship between household technology evolution and the HAGC policy, furnishing insights for more precise strategic interventions. (3) Through rigorous empirical scrutiny, we demystify the direct ramifications of household technological progress on the structure of children’s nutritional intake. Such discernment holds pragmatic value for policymakers, equipping them with actionable insights to refine public health strategies aimed at augmenting the quality of life for children in rural precincts.

The remainder of this paper will be organized as follows. The second part describes possible impacts of household appliances on children’s nutritional intake. The third section describes the data sources, the variables required for the empirical analysis, and the research methodology. Part four presents and discusses the findings. The last part draws conclusions from this study.

## 2 Background

### 2.1 The background of “home appliances going to the countryside” policy

In 2007, the global financial crisis, emanating from the United States and reverberating globally, markedly impacted China’s economy, with the household appliance sector being especially affected. In response, the Chinese government inaugurated the “Home Appliances Going to the Countryside” initiative in December 2007. Initially, this policy was trialed in three provinces: Shandong, Henan, and Sichuan, as well as Qingdao city, spotlighting key pilot products like refrigerators, mobile phones, and color televisions. To incentivize purchases, a 13% subsidy was extended to consumers of these products. By December 2008, the initiative had been broadened to encompass 11 additional provinces, including Hunan, Guangxi, and Chongqing, among others, amplifying the household appliance subsidy’s reach in rural locales. By 2009, nine distinct product categories fell under the policy’s purview, with nationwide coverage. Further bolstering the initiative, a 2009 government directive elevated the maximum price caps for various appliances and instituted fixed subsidies for products surpassing these thresholds. For illustration, the price ceiling for color televisions surged from 3500 yuan to 7000 yuan, accompanied by a fixed 455 yuan subsidy for each unit surpassing the initial 3500 yuan threshold.^1^ Cumulatively, by December 2012, the program facilitated the sale of approximately 298 million household appliance units, yielding a revenue of 720.4 billion yuan.^2^ The policy culminated in early 2013, having effectively galvanized rural consumption and mitigated economic hardships.

According to the research of existing literature (9), refrigerators, washing machines and color TVs are the research objects in this paper. Our emphasis on these appliances is threefold. Firstly, the subsidy for these items was uniform nationally, bestowing a 13 percent concession to consumers. Secondly, the China Health and Nutrition Survey (CHNS) data predominantly offers ownership information pertinent to these appliances. Lastly, the inherent linkage between these appliances and children’s nutritional intake is palpable. For instance, color televisions can serve as conduits for nutritional information dissemination, while washing machines, by virtue of time-saving, can indirectly facilitate more nutritious meal preparations by parents. The ensuing sections elucidate this conceptual framework in depth.

**FIGURE 1:**
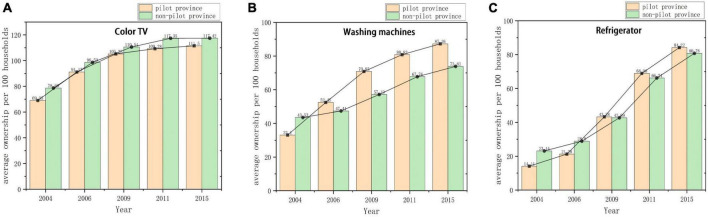
Average ownership of household appliances in pilot and non-pilot provinces before and after HAGC policy implementation. **(A)** Color TV; **(B)** Washing machine; **(C)** Television.

The introduction of the HAGC policy markedly influenced the uptake of household appliances within rural regions. As shown in Figures 1A–C, there’s a discernible escalation in the average quantity of household appliances (Color TV, Washing machines, Refrigerator) per hundred households when contrasting pilot areas with non-pilot regions, both prior to and subsequent to the HAGC policy’s rollout.^3^ Notably, post-policy enactment, rural households within the pilot provinces witnessed a pronounced augmentation in their appliance counts. Given its orientation toward benefiting rural demographics, the HAGC policy’s execution offers an opportune quasi-natural experimental setting for our analysis. Within this framework, rural children from pilot provinces are designated as the treatment cohort, while their counterparts from non-pilot provinces constitute the control set. By juxtaposing the variances in nutritional intake architectures between these two cohorts, pre- and post-policy introduction, our objective is to discern the ramifications of household technological advancements on children’s dietary nutrition.

### 2.2 Conceptual framework

**FIGURE 2:**
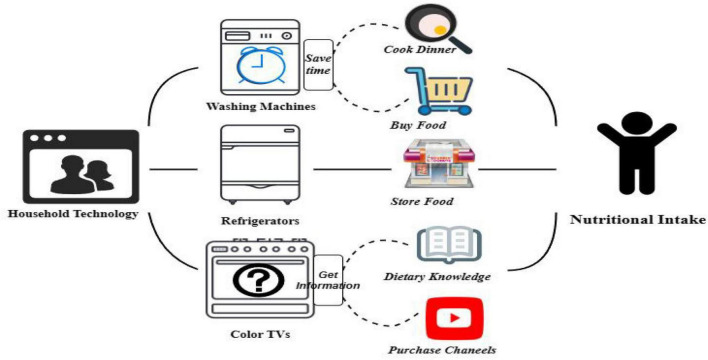
How household technology affects children’s nutritional intake.

This section elucidates the ramifications of the Household technology on the nutritional intake of children in rural areas. As depicted in Figure 2, we present a “Household technology-three home appliances-children’s nutritional intake” model, and subsequently expound upon how each of these three appliances influences children’s nutritional patterns.

To begin, the initiation of the HAGC policy notably accelerates the procurement of washing machines, refrigerators, and color TVs by rural households. Prevailing research corroborates that the enhanced utilization of such household appliances can markedly curtail domestic labor time (12). Specifically, washing machines, recognized as a predominant “time-saving” appliance (13), can appreciably augment leisure time by streamlining chores (14). Consequently, rural family members can channel this extra time into purchasing and preparing nutrient-rich meals for children. Moreover, for women, this technology-driven time optimization has refined their time management—gravitating more toward market visits than mundane domestic tasks (12), thereby enabling better nutritional choices for their children and indirectly ameliorating the prevalence of child malnutrition in rural areas.

Refrigerators, quintessential household items, not only proffer extended food preservation but also alleviate domestic responsibilities for women (15). Refrigerators play a pivotal role in safeguarding the shelf life of nutrient-rich yet perishable foods such as fish, meats, and frozen commodities (16). This amenity not only ensures food security but also reduces shopping trips, granting women additional time to concoct nutritious meals. As depicted in the succeeding figure, refrigerators indubitably influence children’s nutritional intake.

Color TVs, denoted as “time-consuming” commodities (17), while occupying discretionary time, augment informational accessibility (18). Owing to the rudimentary infrastructure in rural regions, televisions emerge as primary conduits for nutritional education. Empirical studies confirm media, especially television, as a preeminent source of nutritional guidance (19). When assessing macronutrient assimilation, knowledge acquisition augments women’s intake of protein, fat, and carbohydrates by 2.71, 6.57, and 2.14% respectively (20). Furthermore, televisions also serve as gateways for procuring nutritional goods in these regions, thus indirectly shaping children’s nutritional uptake.

In summation, the adoption of refrigerators and color TVs acts as catalysts in bolstering children’s nutritional consumption in rural settings. A refrigerator is indispensable in rural households, serving as a linchpin in enhancing children’s dietary health through secure and sustained storage of nutritious foods.

## 3 Materials and methods

### 3.1 Data sources

The present study utilized data sourced from the China Health and Nutrition Survey (CHNS),^4^ a longitudinal surveillance system. This survey offers an encompassing insight into China’s evolving trends, encompassing nine provinces distinguished by diverse geographies, economic progress, and health metrics (21, 22). CHNS stands as an international collaborative endeavor jointly orchestrated by the University of North Carolina and the National Institute of Nutrition and Health, under the aegis of the Chinese Center for Disease Control and Prevention (23). Encompassing an array of research domains, the initiative seeks to evaluate the ramifications of health, nutrition, and family planning policies introduced by both national and local administrative bodies. Furthermore, it has been conceptualized to discern how the ongoing socio-economic metamorphosis within Chinese society has influenced the health and nutritional profiles of its citizenry.

The CHNS encompasses a comprehensive dataset spanning the entirety of China, further categorized into eastern, central, and western regions. In this study, a methodical multistage, random clustering approach was employed, yielding a sample of 2,910 children across 12 provinces: Guangxi, Guizhou, Henan, Hubei, Hunan, Jiangsu, Liaoning, Shandong, Heilongjiang, Beijing, Shanghai, and Chongqing. Within the CHNS dataset, meticulous records of both familial and individual characteristics are maintained, alongside individual dietary intake spanning a continuous 3-day period. Additionally, the data integrates household food consumption metrics. When synthesizing these elements, researchers are well-equipped to undertake a comprehensive analysis of the nutritional intake among China’s rural child population (24).

The dietary data analyzed in this study were derived from individual self-reports documented over a span of three consecutive days. This encompassed comprehensive inventories, purchasing records, and household weight and measurement data captured both at the outset and conclusion of each day (22). For the purposes of this study, carbohydrate, fat, and protein intakes were evaluated as dependent variables, with their proportions being calculated based on the available data. Given the introduction of the HAGC policy in 2007, our research sourced data from three distinct waves: 2006, 2009, and 2011. Herein, the 2006 dataset serves as a pre-policy implementation benchmark, while datasets from 2009 and 2011 offer post-policy insights. In essence, the CHNS stands as a robust reference for discerning causative effects. For a more comprehensive understanding of CHNS, readers are directed to the article authored by Popkin et al. (23).

### 3.2 Variables

#### 3.2.1 Explained variable

Drawing upon established literature (20, 24, 25), this study designates children’s nutritional intake as its primary explanatory variable. Specifically, we focus on three cardinal macronutrients: proteins, fats, and carbohydrates. The centrality of these macronutrients lies in their pivotal role in human energy intake regulation, underscoring their fundamental importance to human sustenance (26, 27). Adequate daily intake of these macronutrients can not only foster optimal growth but also ameliorate conditions of undernutrition in children. Recognizing the inherent heterogeneity across age groups, this study employs the energy intake rates of these nutrients as the definitive explanatory metrics.


(1) $$F\,o\,o\,d_{C}=\frac{C\,a\,r\,b\,o\,h\,y\,d\,r\,a\,t\,e\,s}{C\,a\,r\,b\,o\,h\,y\,d\,r\,a\,t\,e\,s+F\,a\,t\,s+P\,r\,o\,t\,e\,i\,n\,s}$$

(2) $$F\,o\,o\,d_{F}=\frac{F\,a\,t\,s}{C\,a\,r\,b\,o\,h\,y\,d\,r\,a\,t\,e\,s+F\,a\,t\,s+P\,r\,o\,t\,e\,i\,n\,s}$$

(3) $$F\,o\,o\,d_{P}=\frac{P\,r\,o\,t\,e\,i\,n\,s}{C\,a\,r\,b\,o\,h\,y\,d\,r\,a\,t\,s+F\,a\,t\,s+P\,r\,o\,t\,e\,i\,n\,s}$$

#### 3.2.2 Core explanatory variable

Within the Difference-in-Differences (DID) approach adopted in this study, a pivotal component is the interaction term. Our rationale for employing this interaction term is twofold. First, the HAGC policy was specifically oriented toward pilot provinces. The binary variable “Treat” designates whether a province was encompassed by the HAGC policy—with a value of 1 indicating inclusion as a pilot province, and 0 otherwise. Second, the HAGC policy saw expansive promotion in 2009, prompting the introduction of another binary variable, “Post,” which delineates if the survey year is 2009 or subsequent. Here, a value of 1 indicates a survey year of 2009 or later, while 0 represents earlier years. Consequently, the interaction term “Treat × Post” was formulated to capture the policy’s direct influence on a child’s family within the designated timeline.

#### 3.2.3 Control variables

Building on insights from the extant literature (20, 24, 25, 28), this research incorporates control variables spanning three domains: characteristics of the child, parental attributes, and household features. Prevailing evidence indicates that these domains hold significant sway over children’s nutritional intake. Within these domains: factors such as education, sleep duration, self-perceived weight, and health status pertain to children; parents’ educational attainment, time spent cooking, and smoking habits relate to parental attributes; and aspects like water source, ownership of a motorbike, and overall household income are aligned with household features. A comprehensive list of these variables, accompanied by their detailed definitions, is presented in Table 1.

**TABLE 1:** Variables and definitions.

Variables	Abbreviation	Description
**Dependent variables**		
Proportion of carbohydrate intake	food_C	Continuous variable
Proportion of fat intake	food_F	Continuous variable
Proportion of protein intake	food_P	Continuous variable
**Independent variables**		
Whether the province was covered by the HAGC policy	Treat	1 = pilot province; otherwise = 0
Whether the survey year was 2009 or later	Post	1 = 2009 or later; otherwise = 0
**Control variables**		
Sleep time in hours	Sleep time	Continuous variable
Child education in years	Education	Continuous variable
Children’s perception of their own weight	Weight_self	1 = underweight; 2 = normal; 3 = overweight
Parents’ cook time in hours	P_C time	Continuous variable
Been sick or injured in last 4 weeks	Sick	Continuous variable
Parental average education in years	P_Education	Continuous variable
Do parents smoke	P_Smoke	1 = at least one parent smoked; 0 = otherwise
Household income	Ln (income)	Continuous variable, inflated to Chinese CNY in 2015
Whether the household owns motorbike	Motorbike	1 = own motorbike; 0 = otherwise
Tap water availability	Water	1 = available tap water; 0 = otherwise
**Path variables**		
BMI scores	BMI	Continuous variable
Parents’ other time	P_O time	Continuous variable
Color TV ownership	Color TV	1 = Own color TV; 0 = otherwise
Washing machine ownership	Washing machine	1 = Own washing machine; 0 = otherwise
Refrigerator ownership	Refrigerator	1 = Own Refrigerator; 0 = otherwise
On a diet last year	Diet	1 = diet for weight increase; 2 = diet for weight decrease; 0 = otherwise

As depicted in Table 2, the year of the HAGC policy’s introduction serves as a delineation, enabling a structured assessment of core variables between pilot and non-pilot provinces. Table 2 provides comprehensive details on dependent, control, and path variables. After filtering out respondents with incomplete data, a total of 2,910 rural children were retained as the analytical sample for this study.

**TABLE 2:** Summary statistics for the crucial variables.

	Before				After			
	**Pilot provinces**		**Non-pilot provinces**		**Pilot provinces**		**Non-pilot provinces**	
	**Mean**	**SD**	**Mean**	**SD**	**Mean**	**SD**	**Mean**	**SD**
**Dependent variable**								
Food_C	0.73	0.09	0.70	0.08	0.69	0.09	0.67	0.09
Food_F	0.12	0.08	0.16	0.07	0.15	0.09	0.18	0.07
Food_P	0.15	0.03	0.14	0.03	0.16	0.03	0.16	0.04
**Control variables**								
Education	16.93	5.59	17.18	5.65	16.72	5.18	16.89	5.80
Sleep time	9.49	1.46	9.51	1.62	9.36	1.71	9.64	1.75
Weight-self	2.27	1.46	2.04	1.24	2.15	1.22	2.26	1.64
Sick	0.12	0.69	0.09	0.42	0.06	0.25	0.11	0.38
Motorbike	0.56	0.50	0.46	0.50	0.53	0.50	0.56	0.50
Ln (income)	9.64	1.03	9.93	0.93	10.10	1.18	10.52	0.95
Water	0.44	0.50	0.72	0.45	0.70	0.46	0.80	0.40
P-cooktime	0.98	0.84	1.34	0.94	62.91	50.83	73.17	58.72
P-edu	20.09	5.76	20.63	5.48	21.32	5.09	21.20	5.63
P-smoke	0.01	0.10	0.01	0.10	0.00	0.05	0.00	0.06
**Path variables**								
Refrigerator	0.39	0.49	0.42	0.49	0.68	0.47	0.71	0.46
Washing ma∼e	0.74	0.44	0.66	0.47	0.92	0.27	0.78	0.41
TV color	0.92	0.27	0.95	0.22	1.00	0.05	0.98	0.13
P-othertime	82.56	52.84	89.24	70.06	92.00	59.19	97.63	67.29
Diet	0.18	1.15	0.14	0.76	0.34	1.62	0.32	1.35
Insurance	0.30	0.46	0.46	0.50	0.83	0.38	0.88	0.33

### 3.3 Model setting

The central objective of this study is to ascertain the influence of the household technology on the nutritional intake of rural Chinese children. Given the presence of both observable and unobservable factors, pinpointing a direct causal link between household appliances and children’s nutritional intake becomes intricate. Thus, we capitalize on the exogenous price fluctuations triggered by the HAGC policy to establish causality. It’s imperative to note that the HAGC policy operates at a national scale, with its implementation being uniform across regions, rendering it a quasi-natural experimental setting. This study, therefore, assesses the impact of household appliances by juxtaposing children’s nutrient intake metrics before and after the HAGC policy’s inception.

To compare changes in children’s nutritional intake before and after HAGC policy implementation, the function was constructed in the following forms:


(4) $$N\,u\,t\,r\,i\,m\,e\,n\,t_{i\,t}=\gamma_{0}+\gamma_{1}\,T\,r\,e\,a\,t_{i\,t}*P\,o\,s\,t_{i\,t}+\varphi_{1}\,X_{i\,t}+\varepsilon_{i\,t}$$

In the model, nutriment means children’s nutritional intake, and subscript i stands for child and subscript t stands for years. represents whether a province belongs to the pilot province (means the province was covered by the HAGC policy; = 0, otherwise) If the survey year was 2009 or later, we judged = 1; if the survey year was before 2009, we judged = 0. means that the household appliances have an impact on children’s nutritional intake. represents a set of control variables that affect children’s nutrient intake. And ultimately means the residuals.

The application of the Difference-in-Differences (DID) method hinges on two critical preconditions: sample randomness and the adherence to parallel trend assumptions (29, 30). Addressing the first of these, the pilot provinces chosen under the HAGC policy satisfy the randomness criterion. The selection for these pilots is anchored on several factors, including geographical location, prevailing market conditions, and population metrics. It’s essential to note that while the selection process isn’t entirely randomized, its correlation with the nutritional intake of rural children remains minimal. Turning to the second precondition, in an effort to mitigate potential biases in the DID estimation outcomes, we’ve employed the Propensity Score Matching (PSM) approach to create a robust “counterfactual framework.” At the heart of PSM lies the intent to align predicted values optimally. For illustrative purposes, let’s consider province “a” as part of the treatment group. Our strategy then revolves around identifying a province “b” from the control group, ensuring that the defining characteristics of entities within both provinces closely mirror one another. When both “a” and “b” demonstrate analogous propensities to be part of the treatment group, this resonates with the foundational parallel trend assumption.

For this research, PSM was harnessed to delineate the control group. Initially, control variables in pilot provinces underwent logistic regression, yielding propensity scores. Specifically, for any identified individual “a” in the treatment cohort, the nearest neighbor matching technique was employed to identify a counterpart “b” in the control segment, adhering to a 1:1 matching criterion. Subsequent steps encompassed validation of the common support hypothesis, ensuring negligible variances between the experimental and control group covariates post-matching. If congruencies were evident, it bolstered the matching quality, and samples aligned with the common support hypothesis were deemed fit for DID regression.

Chronologically, Henan and Shandong provinces pioneered the HAGC policy toward late 2007. The policy’s purview expanded to 14 provinces by 2008, making its influence palpable. From 2009 onward, all provinces were encompassed by the HAGC policy. Given the intrinsic lags of the CHNS survey, data from 2009 reflected the 2008 scenario, and the 2011 data captured 2010. Thus, CHNS data remained unaffected by the HAGC policy until 2009, with post-2011 data being influenced by it. Utilizing the PSM-DID technique, we gauged the impact of household appliances on nutritional intake, contrasting pilot and non-pilot provinces circa 2009. The estimates for the PSM-DID model were used as follows:


(5) p(X)=Pr[D=1|X]

(6) $$N\,u\,t\,r\,i\,m\,e\,n\,t_{i\,t}=\gamma_{0}+\gamma_{1}\,P\,i\,l\,o\,t_{i\,t}*A\,f\,t\,e\,r_{i\,t}+\varphi_{1}\,X_{i\,t}+\varepsilon_{i\,t}$$

In the second formula, D meant all cities of control and experimental groups and X meant control variables. indicated whether the province where the child lives was a pilot province. (= 1 meant the province where child lives was the pilot province; = 0, otherwise) = 1 represents the year of 2009 (after all provinces had been covered by the HAGC policy) and = 0 represents the year of 2006.

## 4 Results

### 4.1 Summary statistics

**FIGURE 3:**
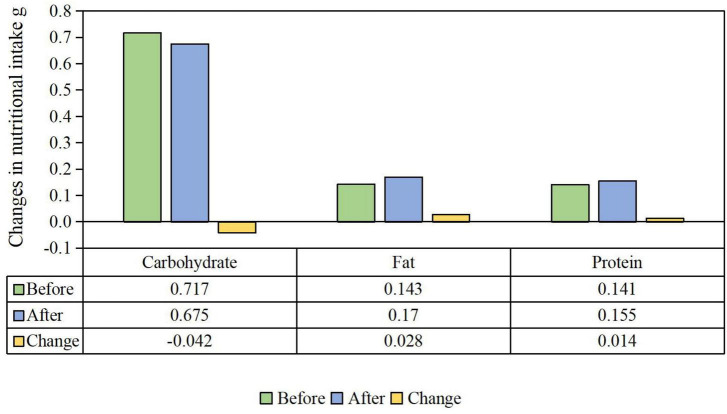
Changes in children’s nutritional intake after the HAGC policy.

As shown in Figure 3, children’s nutritional intake was apparently affected by the implementation of HAGC policy. In detail, children’s carbohydrates intake obviously decreased by 4.2% after the HAGC policy implementation. On the contrary, children’s intake of fats and proteins, respectively, increased by 2.8, 1.4% after the implementation of HAGC policy.

### 4.2 PSM-DID method analysis

#### 4.2.1 Matching effect analysis

Within our study’s framework, we utilized the logit model to determine the matching scores across 12 provinces. Established propensity matching techniques, such as Nearest Neighbor Matching, Mahalanobis distance radius matching, and kernel matching, have been consistently applied in prior research. For the purposes of this study, we predominantly employed the 1:1 nearest neighbor matching approach to delineate our control and treatment groups. In an endeavor to enhance sample compatibility, 11 salient variables were chosen as matching features. These encompassed Age, BMI, Education, Sleep Duration, Parental Education, Parental Cooking Duration, Water Accessibility, Family Income, Available Vehicles, and Household Smoking Habits. Additionally, both radius matching and kernel matching techniques were earmarked for robustness assessment.

#### 4.2.2 Balance test

**FIGURE 4:**
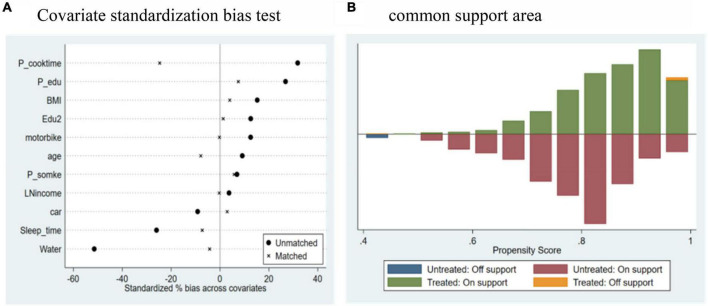
Balance test for matching samples. **(A)** Outcomes of the covariate standardization bias test. **(B)** Propensity score distribution for the treatment and control groups.

To ensure the validity of our matching outcomes, we conducted a rigorous balance test on the matched results. Figure 4A illustrates the outcomes of the covariate normalized bias test. It is clear that, with the exception of Age and P_cooktime, the hypothesis that there is no systematic bias in the values of covariates between the two groups was not rejected for the remaining covariates. Figure 4B, through a histogram, provides a graphical representation of the sample distribution. Notably, in Figure 4B, a predominant number of samples from both the treatment and control groups were observed to align within the common value parameters. However, a marginal set of samples exhibited extreme propensity score values, situating them outside of this common range.

#### 4.2.3 Regression results

Informed by the preceding evaluations, we conducted our core regression analysis using the subset of samples that adhere to the common support hypothesis. Table 3 displays the foundational regression estimations. Columns (2), (3), and (4) lucidly delineate the influence of the household technology on varied dimensions of children’s nutritional consumption. The coefficient linked with Treat × Post affirms that the household technology exerted significant ramifications across three distinct dependent variables. In the context of Food_C, the HAGC policy’s initiation corresponded to a marked diminution in children’s carbohydrate intake, evidenced by a coefficient of 0.027, significant at the 1% threshold. Specifically, the adoption of the household technology initiative corresponded to an elevation in children’s fat consumption proportions, registering a coefficient of 0.019, significant at the 5% threshold. Moreover, the household technology was instrumental in augmenting the proportion of children’s protein consumption, affirmed by a coefficient of 0.008, also significant at the 5% threshold.

**TABLE 3:** Estimation results of PSM-DID model.

Variables	food_carbohydrate	food_Fat	food_Protein
Treat × Post	−0.027*** (0.009)	0.019** (0.008)	0.008** (0.004)
Education	0.002* (0.001)	−0.002 (0.001)	−0.001 (0.000)
Sleep_time	−0.004 (0.004)	0.004 (0.003)	−0.001 (0.001)
weight_self	−0.002 (0.003)	0.001 (0.002)	0.001 (0.001)
sick	−0.009 (0.012)	0.009 (0.011)	−0.000 (0.005)
Motorbike	0.009 (0.009)	−0.003 (0.008)	−0.006* (0.003)
Ln (income)	−0.004 (0.005)	0.005 (0.004)	−0.001 (0.002)
Water	0.005 (0.011)	−0.002 (0.010)	−0.003 (0.004)
P_cooktime	0.000 (0.000)	−0.000* (0.000)	0.000 (0.000)
P_education	0.002* (0.001)	−0.002 (0.001)	−0.000 (0.000)
P_smoke	0.033 (0.070)	−0.051 (0.062)	0.018 (0.028)
Constant	0.694*** (0.063)	0.122** (0.056)	0.184*** (0.025)
Observations	1,713	1,713	1,713
R-squared	0.039	0.033	0.034

Standard errors in parentheses;

***p < 0.01,

**p < 0.05,

*p < 0.1.

According to Table 3, several control variables seemingly interplay with children’s nutritional patterns in rural contexts. Echoing findings from prior research (24), elevated educational attainment was inversely correlated with carbohydrate consumption, while positively influencing fat and protein intakes. Offspring and guardians with extended educational tenures tend to exhibit healthier dietary habits, thereby enhancing children’s nutritional uptake (31, 32). During the HAGC policy’s operational years, affluence-driven households in rural locales displayed a propensity to acquire motorcycles. Such vehicular accessibility in households positively influenced children’s protein consumption, a relationship that’s statistically significant at the 10% level. In terms of parental attributes, the duration dedicated to cooking by parents manifested as a significant determinant for children’s fat intake, registering statistical significance at the 10% threshold.

### 4.3 Placebo test

To ensure that the outcomes of our research weren’t influenced by other unobservable factors and to confirm the reliability of the household technology effect in the baseline regression, we conducted a placebo test. Drawing from the studies by Li et al. (33, 34), a placebo test was employed, randomly generating the experimental group. Specifically, we formulated a “pseudo” household technology variable and, using this variable, executed 500 estimations based on our baseline model (eq. 1). We then cataloged the estimated coefficients pertaining to the children’s nutritional intake.

**FIGURE 5:**
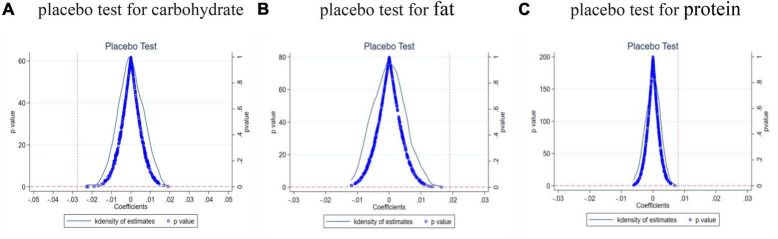
Home Appliances Going to the Countryside (HAGC) policy and children’s nutritional intake placebo test. **(A)** placebo test for children’s carbohydrates intake **(B)** placebo test for children’s fat intake **(C)** placebo test for children’s protein intake.

Figure 5A illustrates the empirical cumulative distribution function and density for the estimated coefficients related to children’s carbohydrate intake. Clearly, after 500 random iterations, the regressions tend to cluster near zero. Reflecting on the regression outcomes detailed in Column 2 of Table 3, the actual baseline estimate was –0.027, markedly lower than the simulated coefficient values. Therefore, it can be inferred that the measurement error associated with the original treatment group in this study is insubstantial. Additionally, employing a consistent methodology, we executed placebo tests for children’s fat and protein intakes. Figures 5B and C sequentially showcase the empirical cumulative distribution function and density for the estimated coefficients concerning children’s fat and protein intakes. These visuals underscore that our assessments are minimally susceptible to disturbances from other arbitrary elements.

### 4.4 Robustness test

To enhance the confidence in our findings regarding the impact of the household technology on children’s nutritional intake, we employed two distinct methods to assess robustness. Initially, we modified the matching technique. While our benchmark regression analysis leveraged the nearest neighbor matching method to align samples and ascertain the optimal control group samples, we explored whether alternative matching approaches would yield varying outcomes. Consequently, both the radius matching and kernel matching methods were employed to corroborate our preliminary conclusions. In addition, our secondary robustness evaluation involved constructing alternative dependent variables. This entailed recalibrating the indicators used for child nutritional intake to validate the stability of our findings.

#### 4.4.1 Choice of matching method

Table 4, as depicted in columns (1) to (3), applied the radius matching method for sample alignment. The derived regression outcomes indicated that the adoption of household technology resulted in a diminished carbohydrate intake in children, while concurrently elevating their fat and protein consumption. This finding mirrored the outcomes obtained through the nearest neighbor matching approach. Columns (4) to (6) of Table 4 employed the kernel matching method for control group sample selection. The regression coefficients related to the HAGC policy variable resonated closely with those of the prior two methodologies, underscoring the robustness of our regression conclusions.

**TABLE 4:** Robustness checks for different matching methods.

	Radius matching			Kernel matching		
**Alternative match methods**	**(1)**	**(2)**	**(3)**	**(4)**	**(5)**	**(6)**
	**food_C**	**food_F**	**food_P**	**food_C**	**food_F**	**food_P**
Treat × Post	−0.038*** (0.009)	0.030*** (0.008)	0.008* (0.004)	−0.031*** (0.011)	0.028*** (0.010)	0.003 (0.004)
Constant	0.736*** (0.056)	0.133*** (0.050)	0.132*** (0.024)	0.681*** (0.065)	0.138** (0.059)	0.181*** (0.026)
Control variables	Y	Y	Y	Y	Y	Y
R-squared	0.058	0.052	0.037	0.039	0.047	0.033
Observations	1,942	1,942	1,942	1,806	1,806	1,806

Standard errors in parentheses:

***p < 0.01,

**p < 0.05,

*p < 0.1.

#### 4.4.2 Substitution of explained variables

Drawing inspiration from prior studies (25, 35), we reconsidered the metrics for children’s nutritional intake, substituting the original dependent variables with measures reflecting carbohydrate, protein, and fat intake. A glance at Table 5 reveals the sustained significance of the core coefficient. The consistency in the results suggests that the explanatory variable substitutions align closely with the estimated outcomes of the original equations (eq. 4). This reinforces the validity of the primary PSM-DID findings, emphasizing that the benchmark regression’s outcomes remain unfazed by variations in the measurement of explained variables.

**TABLE 5:** Robustness checks for substitution of explained variables.

	Alternative explained variables		
	**(1)**	**(2)**	**(3)**
	**Carbohydrate**	**Fat**	**Protein**
Treat × Post	5.958** (2.367)	2.006* (1.122)	2.185*** (0.578)
Constant	36.969* (21.460)	0.022 (10.170)	9.669* (5.236)
Control variables	Y	Y	Y
R-squared	0.077	0.033	0.073
Observations	1,721	1,721	1,721

Standard errors in parentheses:

***p < 0.01,

**p < 0.05,

*p < 0.1.

### 4.5 Heterogeneity analysis

Within certain rural regions of Asia, namely Bangladesh, India, and China, family resources often lean favorably toward boys due to deeply rooted cultural norms (36, 37). China, in particular, showcases these traditional biases, frequently preferring boys over girls in rural settings. This inclination suggests that girls might exhibit heightened sensitivity to nutritional enhancements facilitated by household appliances. Our study’s findings corroborate this perspective. As delineated in Table 6, marked disparities in nutritional intake between boys and girls emerge. Notably, the estimated coefficients for the principal explanatory variables are statistically significant for girls at the 5% level, while they remain non-significant for boys. Such data underscores the probability that advancements in household technology more profoundly impact girls’ nutritional consumption.

**TABLE 6:** Heterogeneity of children’s gender.

	Boys			Girls		
	**(1)**	**(2)**	**(3)**	**(4)**	**(5)**	**(6)**
	**food_C**	**food_F**	**food_P**	**food_C**	**food_F**	**food_P**
Treat × Post	−0.018 (0.014)	0.015 (0.012)	0.003 (0.006)	−0.046** (0.019)	0.044** (0.019)	0.002 (0.007)
Constant	0.678*** (0.081)	0.125* (0.068)	0.197*** (0.034)	0.732*** (0.118)	0.115 (0.114)	0.152*** (0.045)
Control variables	Y	Y	Y	Y	Y	Y
R-squared	0.036	0.037	0.041	0.091	0.102	0.064
Observations	1,028	1,028	1,028	798	798	798

Standard errors in parentheses;

***p < 0.01,

**p < 0.05,

*p < 0.1.

### 4.6 Mechanism analysis

Empirical outcomes from our benchmark regression model illuminate the nuanced impacts of the household technology on children’s consumption of three pivotal macronutrients. To offer a deeper understanding of the interplay between household appliances and children’s nutrient intake, this paper delves into the underlying mechanisms.

A primary channel through which effects manifest is the alteration in parental time allocation attributed to the adoption of household appliances. There’s compelling evidence indicating that the integration of such appliances leads to a reduction in manual tasks, subsequently elevating the time dedicated to childcare (11, 38). To illustrate, the traditional hand-washing of clothes is notably time-intensive for many households; the introduction of washing machines streamlines this process, thereby freeing up time that can be redirected toward preparing nutrient-dense meals for children. This sentiment is corroborated by W You and colleagues, who highlighted parental time allocation as an instrumental environmental determinant of children’s health (39, 40). Our empirical exploration, showcased in Table 7, features positive estimations from core explanatory variables, reinforcing the postulation that the household technology augments parental time for cooking. However, secondary findings suggest that the policy doesn’t notably reshape other facets of parental time. In essence, the household technology amplifies the hours parents allocate for cooking, reaffirming the proposition that it plays a pivotal role in shaping children’s nutritional outcomes.

**TABLE 7:** Mechanism analysis: impact of the HAGC policy on parental time allocation.

	(1)	(2)
**Variables**	**P_cooktime**	**P_othertime**
Treat × Post	40.965*** (6.957)	9.854 (8.325)
Constant	−76.179* (41.773)	133.932*** (49.987)
Control variables	Y	Y
Observations	1,884	1,884
R-squared	0.431	0.036

Standard errors in parentheses:

***p < 0.01,

**p < 0.05,

*p < 0.1.

The integration of household appliances seemingly modulates children’s nutritional intake patterns. Within this scope, we probed the influence of specific appliances—namely, refrigerators, washing machines, and televisions—on the intake of three major macronutrients in children. Referencing Table 8, the utilization of refrigerators notably bolstered the intake of fats and proteins in children, with the significance pegged at the 1% level. This observation finds resonance in existing literature. Martinez et al. postulated a direct correlation between refrigerator usage and augmented protein consumption in humans (41). Likewise, Brent R. and his team supported the notion that the presence of refrigerators in households precipitates a decline in carbohydrate consumption while amplifying the intake of dairy-based nutrients, such as fats and proteins (42). Earlier sections elucidated how washing machines amplify the culinary involvement of parents, serving as an indirect conduit to children’s nutritional profiles. Complementarily, our empirical scrutiny indicated that washing machines curtail children’s carbohydrate consumption while escalating protein intake. Televisions, viewed as prevalent informers of nutritional insights, can potentially shape healthful dietary decisions, thereby enriching children’s nutrient acquisition (43). Existing studies elucidated that television viewing correlates with heightened fat consumption and diminished carbohydrate uptake in adolescents (44). This concurs with the findings presented herein. Moreover, in an era dominated by digital consumption, televisions serve as quintessential portals for e-shoppers to glean product information (39), offering yet another avenue through which they may inadvertently boost children’s nutritional intake.

**TABLE 8:** Mechanism analysis: impact of household appliances on nutrition intake.

	(1)	(2)	(3)
**Variables**	**food_C**	**food_F**	**food_P**
Refrigerator	−0.026*** (0.004)	0.015*** (0.003)	0.011*** (0.002)
Washing_machine	−0.008* (0.004)	0.000 (0.004)	0.007*** (0.002)
TV_color	−0.025** (0.010)	0.016* (0.009)	0.009** (0.004)
Control variables	Y	Y	Y
Observations	2,371	2,371	2,371
R-squared	0.144	0.085	0.136

Standard errors in parentheses;

***p < 0.01,

**p < 0.05,

*p < 0.1.

## 5 Discussion

Our findings underscore the pivotal influence of home appliance adoption on augmenting the nutritional intake among rural children. Altering parental time allocation, optimizing food storage, and enhancing information acquisition emerge as the primary conduits affecting these dietary outcomes. Nevertheless, alternative interpretations might exist. For instance, prolonged food storage could lead to nutrient degradation, particularly concerning fats (40), which might counteract the desired enhancement in children’s nutrition. Moreover, the persuasive power of television advertisements might directly modulate children’s dietary preferences, notably in terms of carbohydrate and fat consumption (45). Research spearheaded by Amanda Avery and collaborators indicates a conspicuous link between television exposure and escalated rates of childhood obesity in developed nations (46, 47). The act of viewing can catalyze passive eating behaviors (48), amplifying the propensity for indulging in snacks and “junk food” (49). Yet, in developing countries, this pattern appears subdued, perhaps due to distinct dietary traditions, constrained food accessibility, and nuances in advertising strategies, among other considerations. Naturally, these observations might display regional or national variations. Such intricacies lie outside the purview of this analysis and present compelling avenues for future scholarly exploration.

Considering home appliances as long-lasting goods, the initiation of the HAGC policy is anticipated to bolster product sales. An uptick in appliance ownership among rural households stands to significantly enhance the family dietary landscape, a step instrumental to elevating children’s nutritional intake. Nonetheless, a potential concern is the undue financial strain this places on rural households, possibly skewing income distribution and, by extension, affecting parental capacity to invest in high-quality ingredients. It becomes imperative for governmental bodies to heighten their focus on the overarching financial health of these households and expand welfare schemes appropriately. Moreover, to foster the sustained growth of the sector, government-subsidy programs should adopt a holistic approach. This implies extending policy support and incentives across the entire home appliance industry value chain. Furthermore, rigorous government oversight remains paramount to preempt the undue leveraging of private interests by corporate entities and individuals.

This study presents certain limitations. Firstly, due to the limitations of CHNS data, it is difficult to determine the influence of other household appliances on children’s nutritional intake. Secondly, the expenditure on household appliances takes up a part of family income, which might impact parents’ assurance of food ingredient quality, and in turn, affect children’s nutritional intake. In the course of our future research, armed with the relevant data and information we acquire, we will strive to address the aforementioned questions. Furthermore, the integration of qualitative methodologies promises to yield a more nuanced understanding of how such policies tangibly influence everyday routines and dietary practices. Such an exploration is vital for deepening our comprehension of the intricate interplay between technology, policy frameworks, and the nutritional health of children within rural contexts.

## 6 Conclusion

Using the eligibility criteria for HAGC subsidies as a basis for a quasi-natural experiment, we explored the influence of household technology on the nuances of children’s nutritional intake. Our investigation encompassed mechanistic considerations such as shifts in parental time allocation, the dynamics of food storage, and the broadened spectrum of information access. By distinguishing between the adoption rates of household appliances in pilot vs. non-pilot provinces, we employed a propensity score matching difference-in-difference approach to discern the ramifications of enhanced household technology on children’s nutritional patterns. Central to our findings was the observation that in the pilot provinces, the integration of household appliances markedly reshaped the nutritional landscape for rural children, manifested by a decreased carbohydrate intake juxtaposed with a heightened intake of fats and proteins.

Furthermore, a layered analysis on the varying impacts of household technology uptake on children’s nutrition was undertaken. A gender-centric dissection revealed a pronounced technological influence on girls, surpassing its effect on boys. To unpack the intricate drivers behind these outcomes, we delved deeper into the spheres of parental time commitments, food preservation, and information accessibility vis-a-vis appliance adoption. The empirical insights from our mechanism analysis synergistically resonated with the preliminary regression findings.

## Data availability statement

The original contributions presented in the study are included in the article/supplementary material, further inquiries can be directed to the corresponding author.

## Author contributions

MD: Conceptualization, Methodology, Software, Writing – original draft. JS: Conceptualization, Software, Writing – review and editing. SS: Visualization, Writing – review and editing. FW: Funding acquisition, Project administration, Supervision, Writing – review and editing.

## Footnotes

1 The implementation plan of the pilot work on rural household appliances issued by the Ministry of Commerce, the Ministry of Finance and the Ministry of Industry and Information Technology of the People’s Republic of China (http://www.mofcom.gov.cn/article/zcfb/zcwg/201002/20100206793004.shtml).

2 Chinese government website, Ministry of Commerce: As of the end of December 2012, the cumulative sales of home appliances to the countryside and sales volume (http://www.mofcom.gov.cn/article/xwfb/xwrcxw/201301/20130108513698.shtml).

3 Per capita ownership of household appliances in rural households in pilot and non-pilot provinces, 2004–2015 (http://www.stats.gov.cn/).

4 CHNS is an ongoing longitudinal data project launched collaboratively by the Carolina Population Center at the University of North Carolina at Chapel Hill and the National Institute of Nutrition and Food Safety at the Chinese Center for Disease Control and Prevention (https://www.cpc.unc.edu/projects/china).
